# Applications of magnesium iodide structure via modified-polynomials

**DOI:** 10.1038/s41598-024-64344-6

**Published:** 2024-06-11

**Authors:** Haleemah Ghazwani, Muhammad Kamran Jamil, Ali Ahmad, Muhammad Azeem, Ali N. A. Koam

**Affiliations:** 1 Department of Mathematics, College of Science, Jazan University, Jazan, 45142 Saudi Arabia; 2 Department of Mathematics, Riphah International University, Lahore, Pakistan; 3 Department of Computer Science, College of Engineering and Computer Science, Jazan University, Jazan, Saudi Arabia

**Keywords:** Algebraic formation, Magnesium iodide, Algebraic properties, Modified polynomials, Topological descriptors, 15A18, 05C90, 05C70, 05C50, 05C10, Biochemistry, Chemistry, Materials science, Mathematics and computing, Physics

## Abstract

A relatively recent approach in molecular graph theory for analyzing chemical networks and structures is called a modified polynomial. It emphasizes the characteristics of molecules through the use of a polynomial-based procedure and presents numerical descriptors in algebraic form. The Quantitative Structure-Property Relationship study makes use of Modified Polynomials (M-Polynomials) as a mathematical tool. M-Polynomials used to create connections between a material’s various properties and its structural characteristics. In this study, we calculated several modified polynomials and gave a polynomial description of the magnesium iodide structure. Particularly, we computed first, second and modified Zagreb indices based M-polynomials. Randić index, and inverse Randić indices based M-polynomials are also computed in this work.

## Introduction

The molecular equation $Mgl_2$ refers to a substance known as magnesium iodide. It has multiple profitable applications and is useful for obtaining various organic syntheses, even though $Mgl_2,$ or magnesium iodide, is an inorganic chemical. The availability measurements of $Mgl_2$ are nanopowder, submicron, high impurity, and volumes. It is a valuable tool in medical practice. Hydro-iodic acid may be used to make magnesium iodide by reacting it with magnesium oxide, magnesium hydroxide, and magnesium carbonate. According to molecular graph theory, the structure of magnesium iodide is a distinct $C_4$-graph arrangement. Multiple heptagons are linked together, each with a $C_4$-graph within. We named the variables as follows for a clearer comprehension of the magnesium iodide molecular graph: The count on the bottom portion $C_4$’s in septagon is *n*,  while the number of upper sides $C_4$’s in a row is *m*. The chemical structure of magnesium iodide is essential to keep the connection of ${\alpha }=2\left( {\beta }+1\right) ,$ and ${\alpha }=2{\beta }+1$ for odd and even amount of *m* individually, for every potential input of ${\beta }\in {\mathbb {Z}}$ with ${\beta }\ge 1$^1–3^.

Modified polynomials are a type of mathematical tool used in quantitative structure-property relationship (QSPR) research. They are used to establish relationships between a molecule’s or material’s structural traits and its numerous attributes. These polynomials are very useful for predicting molecule or material properties based on structural features^4–6^.

Modified polynomials are obtained from the adjacency matrix of the matching graph for a specific molecular or crystal structure. In the example of magnesium iodide (MgI2) crystal structure, the first step would be to create a graph representation of the crystal lattice, having nodes representing atoms (in this case, magnesium and iodine atoms) and edges representing their interactions (chemical bonds). The modified polynomials may then be constructed using the graph’s adjacency matrix^7–9^.

However, computing modified polynomials requires sophisticated mathematical processes and specialized software. The resultant modified polynomials may then be employed in regression analysis to link magnesium iodide structural features with specific attributes of interest, such as electrical, optical, or mechanical properties. It should be noted that the particular shape and values of modified polynomials are determined by the collection of topological indices and descriptors used to define the crystal structure. These indices are chosen depending on the qualities being investigated and the connections being investigated. Ultrahigh specific strength in a magnesium alloy strengthened by spinodal decomposition^10–12^. Comparison of cold sprayed coatings of copper based composite deposited on AZ31B magnesium alloy and 6061 T6 aluminum alloy substrates^13–15^.

Magnesium iodide was chosen for this study due to its significant role in various chemical and industrial applications, including as a catalyst and in organic synthesis. Additionally, its unique structural properties provide an interesting case for analyzing topological descriptors, potentially offering insights that could be applicable to similar compounds. Modified polynomial is more advance technique than topological descriptor. Moreover, this technique can also lead to the topological descriptor. Therefore, we have chosen this work.

We investigated several modified polynomials with magnesium iodide composition in this study for both situations of $\alpha .$ The investigated modified polynomials are specified in Definitions 1.1 to 1.4, along with their other basics, below.

### Preliminaries

In this subsection, we have defined some formulas and some basics of the topics.

#### Definition 1.1

^16^

“Hosoya polynomials, the most well-known and first, were initially released by^17^ in 1988, and modified polynomials which are called modified-polynomial were introduced by^16,18^ in 2015. A strong connection exists between this type of polynomial and degree-based topological indices. It is possible to get topological indices for a network from its modified polynomials utilizing a certain syntax. You can express this modified polynomial as the following:

1 $$\begin{aligned} M\left( {G};{x},{y}\right) =\sum _{{i}\le {j}}{\alpha }_{{i},{j}}\left( {G}\right) {x}^{i}{y}^{j}, \end{aligned}$$

where $\alpha _{{i},{j}}\left( {G}\right)$ is considered as the size of graph *G* given that ${i}\le {j}.$”

#### Definition 1.2

The researchers in^19,20^ developed first and second Zagreb index in the year of 1972, while their modified polynomials derived in^18^,as :

2 $$\begin{aligned} M_1({G})&=\sum _{{u}{v} \in E({G})} ({d}_{u}+{d}_{v}), \end{aligned}$$

3 $$\begin{aligned} M_2({G})&=\sum _{{u}{v} \in E({G})} ({d}_{u}\times {d}_{v}), \end{aligned}$$

4 $$\begin{aligned} {P}_{M_1}({G})&=\left( \Delta {x}+\Delta {y}\right) \left( M\left( {G};{x},{y}\right) \right) , \end{aligned}$$

5 $$\begin{aligned} {P}_{M_2}({G})&=\left( \Delta {x}\Delta {y}\right) \left( M\left( {G};{x},{y}\right) \right) . \end{aligned}$$

#### Definition 1.3

The second type of modified Zagreb index developed in the research work of^21^ and modified polynomial derived in^18^ as:

6 $$\begin{aligned} ^{m}M_2({G})&=\sum _{{u}{v} \in E({G})}\frac{1}{{d}_{u}\times {d}_{v}} \end{aligned}$$

7 $$\begin{aligned} {P}_{^{m}M_2}({G})&=\left( S_{x}S_{y}\right) \left( M\left( {G};{x},{y}\right) \right) . \end{aligned}$$

#### Definition 1.4

In 1988, both researchers of^22,23^, put forward the idea of the general Randić index separately. Given below are the formulation of general Randić, and its inverse version. Moreover, their modified polynomials introduced by^18^, formulated as:

8 $$\begin{aligned} R_{\alpha }\left( {G}\right)&=\sum _{uv \in {E}\left( {G}\right) }({d}_{u}\times {d}_{v})^\alpha , \end{aligned}$$

9 $$\begin{aligned} {P}_{R_{\alpha }}({G})&=\left( \Delta {x}^{\alpha }\Delta {y}^{\alpha }\right) \left( M\left( {G};{x},{y}\right) \right) , \end{aligned}$$

10 $$\begin{aligned} IR_{\alpha }({G})&=\sum _{{u}{v} \in E({G})}\left( \frac{1}{{d}_{u}\times {d}_{v}}\right) ^{\alpha }, \end{aligned}$$

11 $$\begin{aligned} {P}_{IR_{\alpha }}({G})&=\left( S_{x}^{\alpha }S_{y}^{\alpha }\right) \left( M\left( {G};{x},{y}\right) \right) . \end{aligned}$$

where

12 $$\begin{aligned} \Delta {x}\left( f\left( {x},{y}\right) \right)&={x}\frac{\partial f\left( {x},{y}\right) }{\partial {x}}, \end{aligned}$$

13 $$\begin{aligned} \Delta {y}\left( f\left( {x},{y}\right) \right)&={x}\frac{\partial f\left( {x},{y}\right) }{\partial {y}}, \end{aligned}$$

14 $$\begin{aligned} S_{x}\left( f\left( {x},{y}\right) \right)&=\int _{0}^{x}\frac{f\left( {z},{y}\right) }{z}d{z}, \end{aligned}$$

15 $$\begin{aligned} S_{y}\left( f\left( {x},{y}\right) \right)&=\int _{0}^{y}\frac{f\left( {x},{z}\right) }{z}d{z}, \end{aligned}$$

16 $$\begin{aligned} {J}\left( f\left( {x},{y}\right) \right)&=f\left( {x},{x}\right) , \end{aligned}$$

17 $$\begin{aligned} Q_{u}\left( f\left( {x},{y}\right) \right)&={x}^{u}f\left( {x},{y}\right) . \end{aligned}$$

For further notations and symbols, we refer to see Table 1.

**Table 1:** Notations and nomenclatures.

*G*	A simple connected graph
*d*(*v*)	Degree of a vertex
*u* *v*	An edge based on both end vertices (*u* and *v*)
*V*	Vertex set
*E*	Edge set
${M}\left( {G}; {x},{y}\right)$	Modified or M-polynomial
$\left( {d}_{u},{d}_{v}\right)$	An edge with both end vertices degree ${d}_{u}$ and ${d}_{u}$
${V}_{i}$	A vertex type based on the degree of the vertices
${E}_{i,j}$	An edge type based on the degree of the both end vertices

### Literature review

The investigators in^18^ proposed the notion of algebraic graph theory using numerical descriptors (known as topological indices^24–27^. The notion is known as graph-modified polynomials. It contains basics derived from topological indices. We refer to the papers^28–31^ for certain fundamentals and essential topological indicators. This topic has been intensively investigated over the previous half-decade, and there is a plethora of information available. Just a handful of the most recent works on the topic will be discussed.

Modified polynomials of metal-organic chemistry networks are investigated in^32,33^, several benzenoid compounds are highlighted in^34^, the probability notion of modified polynomials interacting with statistics is found in^35^. Graph generalization categories and groups are investigated using various modified polynomials in^29,36,37^, and for $VC_5C_7$-type of nanotubes are discussed in^38^, various nanostructures studied in^39^, There is research on modified polynomials on nanomaterials, for h-naphthenic nanotube^40^. The notion of modified polynomials is also investigated for multiple computerized systems and can be identified in the^41–45^.

For the magnesium iodide structure, $Mgl_2$, the modified polynomials of the first, second, modified Zagreb index, general, and inverse Randić index are calculated, for both even and odd occurrences of parameter $\alpha .$

## Main results

Some significant findings from this study project are presented in this section. The structural amount of the $Mgl_2$ graph, or magnesium iodide graph, as stated in the Tables 2 (for ${\alpha }=odd$) and 3 (for ${\alpha }=even$), are completely dependent on the concept. .

**Case 1** With given ${\beta }\ge 1,$ for the odd amount of ${\alpha }.$ Let ${\alpha }=2{\beta }+1$ and ${\beta }\in {\mathbb {Z}}.$

**Figure 1:**
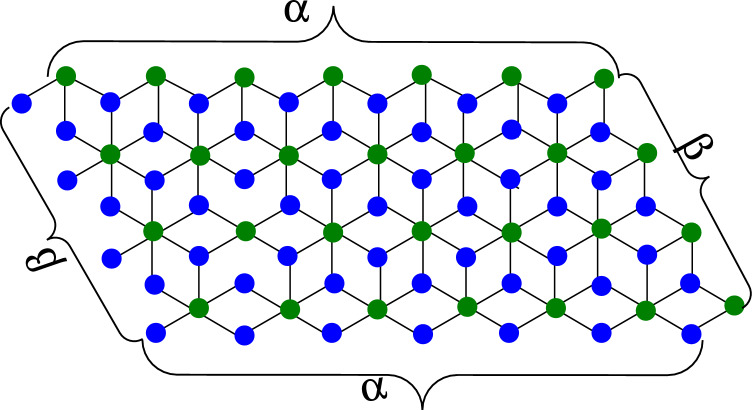
Magnesium Iodide Graph (MIG).

In the Table 2, $({d}_{u},\ {d}_{v})$ is the edge type, which depends on the degree of both end vertices making an edge. Then there is a parameter called frequency which is the count of total edges. For example, there is an edge type (1, 3) there is a single edge of such type whereas one side degree of an edge is one and the other one is three.

**Table 2:** Edge partition of ${MIG}({\alpha },{\beta })$ ${\alpha }=2{\beta }+1$.

$({d}_{u},\ {d}_{v})$	Frequency	Set of edges
(1, 3)	1	$E_{1}$
(1, 4)	1	$E_{2}$
(1, 6)	${\beta }+5$	$E_{3}$
(2, 3)	2	$E_{4}$
(2, 4)	2	$E_{5}$
(2, 5)	8	$E_{6}$
(2, 6)	$2{\beta }+8$	$E_{7}$
(3, 3)	$3{\beta }$	$E_{8}$
(3, 4)	1	$E_{9}$
(3, 5)	12	$E_{10}$
(3, 6)	$27{\beta }-13$	$E_{11}$

### Theorem 2.1

If ${MIG}_{{\alpha },{\beta }}$ be the configuration of magnesium iodide, having ${\alpha }=2{\beta }+1,$ ${\beta }\ge 1,$ appeared in Fig. 1, then, its modified polynomial is:

18 $$\begin{aligned} M\left( {MIG}_{{\alpha },{\beta }},{x},{y}\right)&={x}\left[ {y}^3+{y}^4+\left( {\beta }+5\right) {y}^6\right] + 2{x}^2\left[ {y}^3+{y}^4+4{y}^5+\left( {\beta }+4\right) {y}^6\right] \nonumber \\&\quad + {x}^3\left[ 3{\beta }{y}^3+{y}^4+12{y}^5+\left( 27{\beta }-13\right) {y}^6\right] . \end{aligned}$$

### Proof

The graphical representation of magnesium iodide is built from its molecular makeup, as shown in Fig. 1. According to the picture, there are six types of vertices, and these vertices are specified in the set of vertices that follows:

$$\begin{aligned} {V}_1&=\left\{ {u}\in {V}({MIG}_{{\alpha },{\beta }}): {{d}}_{u}=1\right\} \\ {V}_2&=\left\{ {u}\in {V}({MIG}_{{\alpha },{\beta }}): {{d}}_{u}=2\right\} \\ {V}_3&=\left\{ {u}\in {V}({MIG}_{{\alpha },{\beta }}): {{d}}_{u}=3\right\} \\ {V}_4&=\left\{ {u}\in {V}({MIG}_{{\alpha },{\beta }}): {{d}}_{u}=4\right\} \\ {V}_5&=\left\{ {u}\in {V}({MIG}_{{\alpha },{\beta }}): {{d}}_{u}=5\right\} \\ {V}_6&=\left\{ {u}\in {V}({MIG}_{{\alpha },{\beta }}): {{d}}_{u}=6\right\} . \end{aligned}$$

The identical graphic also included details regarding its edge kinds. It has eleven distinct kinds of edges in all, which are characterized follows:

$$\begin{aligned} {E}_{1,3}&=\left\{ {u}{v}\in E({MIG}_{{\alpha },{\beta }}): {{d}}_{u}=1, {{d}}_{v}=3\right\} \\ {E}_{1,4}&=\left\{ {u}{v}\in E({MIG}_{{\alpha },{\beta }}): {{d}}_{u}=1, {{d}}_{v}=4\right\} \\ {E}_{1,6}&=\left\{ {u}{v}\in E({MIG}_{{\alpha },{\beta }}): {{d}}_{u}=1, {{d}}_{v}=6\right\} \\ {E}_{2,3}&=\left\{ {u}{v}\in E({MIG}_{{\alpha },{\beta }}): {{d}}_{u}=2, {{d}}_{v}=3\right\} \\ {E}_{2,4}&=\left\{ {u}{v}\in E({MIG}_{{\alpha },{\beta }}): {{d}}_{u}=2, {{d}}_{v}=4\right\} \\ {E}_{2,5}&=\left\{ {u}{v}\in E({MIG}_{{\alpha },{\beta }}): {{d}}_{u}=2, {{d}}_{v}=5\right\} \\ {E}_{2,6}&=\left\{ {u}{v}\in E({MIG}_{{\alpha },{\beta }}): {{d}}_{u}=2, {{d}}_{v}=6\right\} \\ {E}_{3,3}&=\left\{ {u}{v}\in E({MIG}_{{\alpha },{\beta }}): {{d}}_{u}=3, {{d}}_{v}=3\right\} \\ {E}_{3,4}&=\left\{ {u}{v}\in E({MIG}_{{\alpha },{\beta }}): {{d}}_{u}=3, {{d}}_{v}=4\right\} \\ {E}_{3,5}&=\left\{ {u}{v}\in E({MIG}_{{\alpha },{\beta }}): {{d}}_{u}=3, {{d}}_{v}=5\right\} \\ {E}_{3,6}&=\left\{ {u}{v}\in E({MIG}_{{\alpha },{\beta }}): {{d}}_{u}=3, {{d}}_{v}=6\right\} . \end{aligned}$$

These edge partitions’ cardinality is $\left| {E}_{1,3}\right| =\left| {E}_{1,4}\right| =\left| {E}_{3,4}\right| =1,\left| {E}_{2,3}\right| =\left| {E}_{2,4}\right| =2, \left| {E}_{1,6}\right| ={\beta }+5, \left| {E}_{2,5}\right| =8, \left| {E}_{2,6}\right| =2\left( {\beta }+4\right) , \left| {E}_{3,3}\right| =3{\beta }, \left| {E}_{3,5}\right| =12,$ and $\left| {E}_{3,6}\right| =27{\beta }-13.$ Then, using the description of 1.1, compute the modified polynomial of ${MIG}_{{\alpha },{\beta }}$ is:

19 $$\begin{aligned} M\left( {MIG}_{{\alpha },{\beta }};{x},{y}\right)&=\sum _{{i}\le {j}}{\alpha }_{{i},{j}}\left( {{MIG}_{{\alpha },{\beta }}}\right) {x}^{i}{y}^{j}\nonumber \\&=\left| {E}_{1,3}\right| \left( {{MIG}_{{\alpha },{\beta }}}\right) x^1y^3 + \left| {E}_{1,4}\right| \left( {{MIG}_{{\alpha },{\beta }}}\right) x^1y^4 + \left| {E}_{1,6}\right| \left( {{MIG}_{{\alpha },{\beta }}}\right) x^1y^6 \nonumber \\&\quad + \left| {E}_{2,3}\right| \left( {{MIG}_{{\alpha },{\beta }}}\right) x^2y^3 + \left| {E}_{2,4}\right| \left( {{MIG}_{{\alpha },{\beta }}}\right) x^2y^4 + \left| {E}_{2,5}\right| \left( {{MIG}_{{\alpha },{\beta }}}\right) x^2y^5 \nonumber \\ &\quad + \left| {E}_{2,6}\right| \left( {{MIG}_{{\alpha },{\beta }}}\right) x^2y^6 + \left| {E}_{3,3}\right| \left( {{MIG}_{{\alpha },{\beta }}}\right) x^3y^3 + \left| {E}_{3,4}\right| \left( {{MIG}_{{\alpha },{\beta }}}\right) x^3y^4 \nonumber \\ &\quad + \left| {E}_{3,5}\right| \left( {{MIG}_{{\alpha },{\beta }}}\right) x^3y^5 + \left| {E}_{3,6}\right| \left( {{MIG}_{{\alpha },{\beta }}}\right) x^3y^6,\nonumber \\&={x}\left[ {y}^3+{y}^4+\left( {\beta }+5\right) {y}^6\right] + 2{x}^2\left[ {y}^3+{y}^4+4{y}^5+\left( {\beta }+4\right) {y}^6\right] \nonumber \\ &\quad + {x}^3\left[ 3{\beta }{y}^3+{y}^4+12{y}^5+\left( 27{\beta }-13\right) {y}^6\right] . \end{aligned}$$

$$\square$$

### Lemma 2.1

If ${MIG}_{{\alpha },{\beta }}$ be the configuration of magnesium iodide, having ${\alpha }=2{\beta }+1,$ ${\beta }\ge 1,$ then the differential operators are

20 $$\begin{aligned} \Delta {x}\left[ M\left( {MIG}_{{\alpha },{\beta }};{x},{y}\right) \right]&={x}\left[ {y}^3+{y}^4+\left( {\beta }+5\right) {y}^6\right] + 4{x}^2\left[ {y}^3+{y}^4+4{y}^5+\left( {\beta }+4\right) {y}^6\right] \nonumber \\&\quad + 3{x}^3\left[ 3{\beta }{y}^3+{y}^4+12{y}^5+\left( 27{\beta }-13\right) {y}^6\right] . \end{aligned}$$

21 $$\begin{aligned} \Delta {y}\left[ M\left( {MIG}_{{\alpha },{\beta }};{x},{y}\right) \right]&={x}\left[ 3{y}^3+4{y}^4+6\left( {\beta }+5\right) {y}^6\right] + 2{x}^2\left[ 3{y}^3+4{y}^4+20{y}^5+3\left( 2{\beta }+8\right) {y}^6\right] \nonumber \\&\quad + {x}^3\left[ 9{\beta }{y}^3+4{y}^4+60{y}^5+6\left( 27{\beta }-13\right) {y}^6\right] . \end{aligned}$$

### Proof

Differentiate equation (18) in terms of *x* and then product the attained value with *x*,  nw we have the differential operator $\Delta {x}\left[ M\left( {MIG}_{{\alpha },{\beta }};{x},{y}\right) \right] .$ Similarly, differentiate equation (18) in terms of *y* and now this time product the answer with *y*,  after simplification we will have the required operator value $\Delta {y}\left[ M\left( {MIG}_{{\alpha },{\beta }};{x},{y}\right) \right] .$ $\square$

### Lemma 2.2

If ${MIG}_{{\alpha },{\beta }}$ be the configuration of magnesium iodide, having ${\alpha }=2{\beta }+1,$ ${\beta }\ge 1,$ then the integral operators are

22 $$\begin{aligned} S_{x}\left[ M\left( {MIG}_{{\alpha },{\beta }};{x},{y}\right) \right]&={x}\left[ {y}^3+{y}^4+\left( {\beta }+5\right) {y}^6\right] + {x}^2\left[ {y}^3+{y}^4+4{y}^5+\left( {\beta }+4\right) {y}^6\right] \nonumber \\&\quad + \frac{1}{3}{x}^3\left[ 3{\beta }{y}^3+{y}^4+12{y}^5+\left( 27{\beta }-13\right) {y}^6\right] . \end{aligned}$$

23 $$\begin{aligned} S_{y}\left[ M\left( {MIG}_{{\alpha },{\beta }};{x},{y}\right) \right]&={x}\left[ \frac{1}{3}{y}^3+\frac{1}{4}{y}^4+\frac{\left( {\beta }+5\right) }{6}{y}^6\right] + {x}^2\left[ \frac{2}{3}{y}^3+\frac{1}{2}{y}^4+\frac{8}{5}{y}^5+\frac{\left( {\beta }+4\right) }{3}{y}^6\right] \nonumber \\&\quad + {x}^3\left[ {\beta }{y}^3+\frac{1}{4}{y}^4+\frac{12}{5}{y}^5+\frac{\left( 27{\beta }-13\right) }{6}{y}^6\right] . \end{aligned}$$

### Proof

It is given in the equation (14), $S_{x}\left[ M\left( {MIG}_{{\alpha },{\beta }};{x},{y}\right) \right] =\int _{0}^{x}\frac{M\left( {MIG}_{{\alpha },{\beta }};{t},{y}\right) }{t}dt.$ Now evaluate the equation (18) which is the methodology of the general modified polynomial for the structure of ${MIG}_{{\alpha },{\beta }}.$ Following a few mathematical calculations, we will have $S_{x}\left[ M\left( {MIG}_{{\alpha },{\beta }};{x},{y}\right) \right] .$ Similarly, from equation (15), $S_{y}\left[ M\left( {MIG}_{{\alpha },{\beta }};{x},{y}\right) \right] =\int _{0}^{y}\frac{M\left( {MIG}_{{\alpha },{\beta }};{x},{t}\right) }{t}dt.$ Now evaluate the equation (18) which is the methodology of the general modified polynomial for the structure of ${MIG}_{{\alpha },{\beta }}.$ Following a few mathematical calculations, we will have $S_{y}\left[ M\left( {MIG}_{{\alpha },{\beta }};{x},{y}\right) \right] .$ $\square$

### Lemma 2.3

Let ${MIG}_{{\alpha },{\beta }}$ be the configuration of magnesium iodide, having ${\alpha }=2{\beta }+1,$ ${\beta }\ge 1.$ Then the *J* operator for the equation 18 is

$$\begin{aligned} {J}\left[ M\left( {MIG}_{{\alpha },{\beta }};{x},{y}\right) \right]&={x}^4+3{x}^5+\left( 3{\beta }+2\right) {x}^6+\left( {\beta }+8\right) {x}^7+2\left( {\beta }+10\right) {x}^8+\left( 27{\beta }-13\right) {x}^9. \end{aligned}$$

### Proof

We obtain the by applying the operator mentioned in the equation 16 on the fundamental equation described in 18${J}\left[ M\left( {MIG}_{{\alpha },{\beta }};{x},{y}\right) \right] .$ $\square$

### Theorem 2.2

Let ${MIG}_{{\alpha },{\beta }}$ be the configuration of magnesium iodide, having ${\alpha }=2{\beta }+1,$ ${\beta }\ge 1,$ and ${P}_{M_1}$ and ${P}_{M_2}$ are the “modified polynomial of first and second Zagreb indices”. Then ${P}_{M_1}\left( {{MIG}_{{\alpha },{\beta }}}\right)$ and ${P}_{M_2}\left( {{MIG}_{{\alpha },{\beta }}}\right)$ is

24 $$\begin{aligned} {P}_{M_1}\left( {{MIG}_{{\alpha },{\beta }}}\right)&={x}\left[ 4{y}^3+5{y}^4+7\left( {\beta }+5\right) {y}^6\right] + 2{x}^2\left[ 5{y}^3+6{y}^4+28{y}^5+8\left( {\beta }+4\right) {y}^6\right] \nonumber \\&\quad + {x}^3\left[ 18{\beta }{y}^3+7{y}^4+96{y}^5+9\left( 27{\beta }-13\right) {y}^6\right] . \end{aligned}$$

25 $$\begin{aligned} {P}_{M_2}\left( {{MIG}_{{\alpha },{\beta }}}\right)&={x}\left[ 3{y}^3+4{y}^4+6\left( {\beta }+5\right) {y}^6\right] + 4{x}^2\left[ 3{y}^3+4{y}^4+20{y}^5+3\left( 2{\beta }+8\right) {y}^6\right] \nonumber \\&\quad + 3{x}^3\left[ 9{\beta }{y}^3+4{y}^4+60{y}^5+6\left( 27{\beta }-13\right) {y}^6\right] . \end{aligned}$$

### Proof

The technique of the modified polynomial of first Zagreb index given in the Definition 1.2, this methodology can be written as ${P}_{M_1}({{MIG}_{{\alpha },{\beta }}})=\left( \Delta {x}+\Delta {y}\right) \left( M\left( {{MIG}_{{\alpha },{\beta }}};{x},{y}\right) \right) ,$ for ${MIG}_{{\alpha },{\beta }}$-structure. Now evaluating the differential operators described in Lemma 2.1, for the structure of ${MIG}_{{\alpha },{\beta }}.$ Following a few mathematical calculations we are going to accomplish the desired outcome of modified polynomial of first Zagreb index for ${MIG}_{{\alpha },{\beta }}$ as:

$${P}_{M_1}\left( {{MIG}_{{\alpha },{\beta }}}\right) ={x}\left[ 4{y}^3+5{y}^4+7\left( {\beta }+5\right) {y}^6\right] + 2{x}^2\left[ 5{y}^3+6{y}^4+28{y}^5+8\left( {\beta }+4\right) {y}^6\right] + {x}^3\left[ 18{\beta }{y}^3+7{y}^4+96{y}^5+9\left( 27{\beta }-13\right) {y}^6\right] .$$

Similarly evaluating the differential operators, given in the Lemma 2.1 for the structure of ${MIG}_{{\alpha },{\beta }}$ in ${P}_{M_2}({{MIG}_{{\alpha },{\beta }}})=\left( \Delta {x}\Delta {y}\right) \left( M\left( {{MIG}_{{\alpha },{\beta }}};{x},{y}\right) \right) ,$ and Following a few mathematical calculations, we are going to accomplish the desired outcomes of modified polynomial of second Zagreb index for ${MIG}_{{\alpha },{\beta }}$:

$${P}_{M_2}\left( {{MIG}_{{\alpha },{\beta }}}\right) ={x}\left[ 3{y}^3+4{y}^4+6\left( {\beta }+5\right) {y}^6\right] + 4{x}^2\left[ 3{y}^3+4{y}^4+20{y}^5+3\left( 2{\beta }+8\right) {y}^6\right] + 3{x}^3\left[ 9{\beta }{y}^3+4{y}^4+60{y}^5+6\left( 27{\beta }-13\right) {y}^6\right] .$$ $$\square$$

### Theorem 2.3

Let ${MIG}_{{\alpha },{\beta }}$ be the configuration of magnesium iodide, having ${\alpha }=2{\beta }+1,$ ${\beta }\ge 1,$ and ${P}_{^{m}M_2}$ is the “modified polynomial of second modified Zagreb index”. Then ${P}_{^{m}M_2}\left( {{MIG}_{{\alpha },{\beta }}}\right)$ is

26 $$\begin{aligned} {P}_{^{m}M_2}({{MIG}_{{\alpha },{\beta }}})&={x}\left[ \frac{1}{3}{y}^3+\frac{1}{4}{y}^4+\frac{\left( {\beta }+5\right) }{6}{y}^6\right] + 2{x}^2\left[ \frac{2}{3}{y}^3+\frac{1}{2}{y}^4+\frac{8}{5}{y}^5+\frac{\left( {\beta }+4\right) }{3}{y}^6\right] \nonumber \\&\quad + 3{x}^3\left[ {\beta }{y}^3+\frac{1}{4}{y}^4+\frac{12}{5}{y}^5+\frac{\left( 27{\beta }-13\right) }{6}{y}^6\right] . \end{aligned}$$

### Proof

The technique of the modified polynomial of second modified Zagreb given in the Definition 1.3, this methodology can be written as ${P}_{^{m}M_2}({{MIG}_{{\alpha },{\beta }}})=\left( S_{x}S_{y}\right) \left( M\left( {{MIG}_{{\alpha },{\beta }}};{x},{y}\right) \right) ,$ for ${MIG}_{{\alpha },{\beta }}$-structure. Now evaluating the integral operators described in Lemma 2.2, for the structure of ${MIG}_{{\alpha },{\beta }}.$ Following a few mathematical calculations, we are going to accomplish the desired outcome of modified polynomial of second modified Zagreb index for ${MIG}_{{\alpha },{\beta }}$ as:

$${P}_{^{m}M_2}\left( {{MIG}_{{\alpha },{\beta }}}\right) ={x}\left[ \frac{1}{3}{y}^3+\frac{1}{4}{y}^4+\frac{\left( {\beta }+5\right) }{6}{y}^6\right] + 2{x}^2\left[ \frac{2}{3}{y}^3+\frac{1}{2}{y}^4+\frac{8}{5}{y}^5+\frac{\left( {\beta }+4\right) }{3}{y}^6\right] + 3{x}^3\left[ {\beta }{y}^3+\frac{1}{4}{y}^4+\frac{12}{5}{y}^5+\frac{\left( 27{\beta }-13\right) }{6}{y}^6\right] .$$

$$\square$$

### Theorem 2.4

Let ${MIG}_{{\alpha },{\beta }}$ be the configuration of magnesium iodide, having ${\alpha }=2{\beta }+1,$ ${\beta }\ge 1,$ and ${P}_{R_{\alpha }}$ is the “modified polynomial of general Randić index”. Then ${P}_{R_{\alpha }}\left( {{MIG}_{{\alpha },{\beta }}}\right)$ is

27 $$\begin{aligned} {P}_{R_{\alpha }}({{MIG}_{{\alpha },{\beta }}})&={x}\left[ 3^{\alpha }{y}^3+4^{\alpha }{y}^4+6^{\alpha }\left( {\beta }+5\right) {y}^6\right] + 2^{{\alpha }+1}{x}^2\left[ 3^{\alpha }{y}^3+4^{\alpha }{y}^4+4\times 5^{\alpha }{y}^5\right. \nonumber \\&\quad \left. +6^{\alpha }\left( {\beta }+4\right) {y}^6\right] + 3^{\alpha }{x}^3\left[ 3^{{\alpha }+1}{\beta }{y}^3+4^{\alpha }{y}^4+12\times 5^{\alpha }{y}^5+6^{\alpha }\left( 27{\beta }-13\right) {y}^6\right] . \end{aligned}$$

### Proof

The technique defined in the Definition 1.4 is known as the formula for the modified polynomial of general Randić index for ${MIG}_{{\alpha },{\beta }}$ defined as: ${P}_{R_{\alpha }}({{MIG}_{{\alpha },{\beta }}})=\left( D^{\alpha }_{x}D^{\alpha }_{y}\right) \left( M\left( {{MIG}_{{\alpha },{\beta }}};{x},{y}\right) \right) ,$ by evaluating the formula of generalize version of differential operators given by Lemma 2.1 for ${MIG}_{{\alpha },{\beta }}.$ Following a few mathematical calculations, we are going to accomplish the desired outcomes of modified polynomial of general Randić index for ${MIG}_{{\alpha },{\beta }}$ as:

$${P}_{R_{\alpha }}\left( {{MIG}_{{\alpha },{\beta }}}\right) = {x}\left[ 3^{\alpha }{y}^3+4^{\alpha }{y}^4+6^{\alpha }\left( {\beta }+5\right) {y}^6\right]$$

$$+ 2^{{\alpha }+1}{x}^2\left[ 3^{\alpha }{y}^3+4^{\alpha }{y}^4+4\times 5^{\alpha }{y}^5+6^{\alpha }\left( {\beta }+4\right) {y}^6\right] + 3^{\alpha }{x}^3\left[ 3^{{\alpha }+1}{\beta }{y}^3+4^{\alpha }{y}^4+12\times 5^{\alpha }{y}^5+6^{\alpha }\left( 27{\beta }-13\right) {y}^6\right] .$$ $$\square$$

### Theorem 2.5

Let ${MIG}_{{\alpha },{\beta }}$ be the configuration of magnesium iodide, having ${\alpha }=2{\beta }+1,$ ${\beta }\ge 1,$ and ${P}_{IR_{\alpha }}$ is the “modified polynomial of general inverse Randić index”. Then ${P}_{IR_{\alpha }}\left( {{MIG}_{{\alpha },{\beta }}}\right)$ is

28 $$\begin{aligned} {P}_{IR_{\alpha }}({{MIG}_{{\alpha },{\beta }}})&={x}\left[ \frac{1}{3^{\alpha }}{y}^3+\frac{1}{4^{\alpha }}{y}^4+\frac{\left( {\beta }+5\right) }{6^{\alpha }}{y}^6\right] + \frac{1}{2^{{\alpha }-1}}{x}^2\left[ \frac{1}{3^{\alpha }}{y}^3+\frac{1}{4^{\alpha }}{y}^4+\frac{4}{5^{\alpha }}{y}^5+\frac{\left( {\beta }+4\right) }{6^{\alpha }}{y}^6\right] \nonumber \\&\quad + \frac{1}{3^{\alpha }}{x}^3\left[ \frac{{\beta }}{3^{{\alpha }-1}}{y}^3+\frac{1}{4^{\alpha }}{y}^4+\frac{12}{5^{\alpha }}{y}^5+\frac{\left( 27{\beta }-13\right) }{6^{\alpha }}{y}^6\right] . \end{aligned}$$

### Proof

The technique defined in the Definition 1.4 is known as the formula for the modified polynomial of general inverse version of Randić index for ${MIG}_{{\alpha },{\beta }}$ defined as: ${P}_{IR_{\alpha }}({{MIG}_{{\alpha },{\beta }}})=\left( S^{\alpha }_{x}S^{\alpha }_{y}\right) \left( M\left( {{MIG}_{{\alpha },{\beta }}};{x},{y}\right) \right) ,$ by evaluating the formula of generalize version of integral operators given by Lemma 2.2 for ${MIG}_{{\alpha },{\beta }}.$ Following a few mathematical calculations, we are going to accomplish the desired outcomes of modified polynomial of general inverse version of Randić index for ${MIG}_{{\alpha },{\beta }}$ as:

$${P}_{IR_{\alpha }}\left( {{MIG}_{{\alpha },{\beta }}}\right) = {x}\left[ \frac{1}{3^{\alpha }}{y}^3+\frac{1}{4^{\alpha }}{y}^4+\frac{\left( {\beta }+5\right) }{6^{\alpha }}{y}^6\right] + \frac{1}{2^{{\alpha }-1}}{x}^2\left[ \frac{1}{3^{\alpha }}{y}^3+\frac{1}{4^{\alpha }}{y}^4+\frac{4}{5^{\alpha }}{y}^5+\frac{\left( {\beta }+4\right) }{6^{\alpha }}{y}^6\right]$$

$$+ \frac{1}{3^{\alpha }}{x}^3\left[ \frac{{\beta }}{3^{{\alpha }-1}}{y}^3+\frac{1}{4^{\alpha }}{y}^4+\frac{12}{5^{\alpha }}{y}^5+\frac{\left( 27{\beta }-13\right) }{6^{\alpha }}{y}^6\right] .$$ $$\square$$

**Case 2:** For the even amount of ${\alpha }$ with given ${\beta }\ge 1.$ Let ${\alpha }=2\left( {\beta }+1\right)$ and ${\beta }\in {\mathbb {Z}}.$

**Table 3:** Edge partition of ${MIG}({\alpha },{\beta })$ for ${\alpha }=2\left( {\beta }+1\right)$.

$({d}_{u},\ {d}_{v})$	Frequency	Set of edges
(1, 3)	1	$E_{1}$
(1, 5)	1	$E_{2}$
(1, 6)	${\beta }+5$	$E_{3}$
(2, 2)	2	$E_{4}$
(2, 3)	2	$E_{5}$
(2, 5)	8	$E_{6}$
(2, 6)	$2{\beta }+8$	$E_{7}$
(3, 3)	$3{\beta }$	$E_{8}$
(3, 5)	1	$E_{9}$
(3, 6)	12	$E_{10}$

### Theorem 2.6

Let ${MIG}_{{\alpha },{\beta }}$ be the configuration of magnesium iodide, having ${\alpha }=2\left( {\beta }+1\right) ,$ ${\beta }\ge 1,$ appeared in Fig. 1. Then, its modified polynomial is:

29 $$\begin{aligned} M\left( {MIG}_{{\alpha },{\beta }},x,y\right)&={x}\left[ {y}^3+{y}^5+\left( {\beta }+5\right) {y}^6\right] + {x}^2\left[ 5{y}^2+6{y}^3+2{y}^5+\left( 2{\beta }+8\right) {y}^6\right] \nonumber \\&\quad + {x}^3\left[ \left( 3{\beta }+1\right) {y}^3 + 2{y}^5 + \left( 27{\beta }+7\right) {y}^6\right] . \end{aligned}$$

### Proof

The graphical representation of magnesium iodide is built from its molecular makeup, as shown in Fig. 1. According to the picture, there are six types of vertices, and these vertices are specified in the set of vertices that follows:

$$\begin{aligned} {V}_1&=\left\{ {u}\in {V}({MIG}_{{\alpha },{\beta }}): {{d}}_{u}=1\right\} \\ {V}_2&=\left\{ {u}\in {V}({MIG}_{{\alpha },{\beta }}): {{d}}_{u}=2\right\} \\ {V}_3&=\left\{ {u}\in {V}({MIG}_{{\alpha },{\beta }}): {{d}}_{u}=3\right\} \\ {V}_5&=\left\{ {u}\in {V}({MIG}_{{\alpha },{\beta }}): {{d}}_{u}=5\right\} \\ {V}_6&=\left\{ {u}\in {V}({MIG}_{{\alpha },{\beta }}): {{d}}_{u}=6\right\} . \end{aligned}$$

The identical graphic also included details regarding its edge kinds. It has eleven distinct kinds of edges in all, which are characterized follows:

$$\begin{aligned} {E}_{1,3}&=\left\{ {u}{v}\in E({MIG}_{{\alpha },{\beta }}): {{d}}_{u}=1, {{d}}_{v}=3\right\} \\ {E}_{1,5}&=\left\{ {u}{v}\in E({MIG}_{{\alpha },{\beta }}): {{d}}_{u}=1, {{d}}_{v}=5\right\} \\ {E}_{1,6}&=\left\{ {u}{v}\in E({MIG}_{{\alpha },{\beta }}): {{d}}_{u}=1, {{d}}_{v}=6\right\} \\ {E}_{2,2}&=\left\{ {u}{v}\in E({MIG}_{{\alpha },{\beta }}): {{d}}_{u}=2, {{d}}_{v}=2\right\} \\ {E}_{2,3}&=\left\{ {u}{v}\in E({MIG}_{{\alpha },{\beta }}): {{d}}_{u}=2, {{d}}_{v}=3\right\} \\ {E}_{2,5}&=\left\{ {u}{v}\in E({MIG}_{{\alpha },{\beta }}): {{d}}_{u}=2, {{d}}_{v}=5\right\} \\ {E}_{2,6}&=\left\{ {u}{v}\in E({MIG}_{{\alpha },{\beta }}): {{d}}_{u}=2, {{d}}_{v}=6\right\} \\ {E}_{3,3}&=\left\{ {u}{v}\in E({MIG}_{{\alpha },{\beta }}): {{d}}_{u}=3, {{d}}_{v}=3\right\} \\ {E}_{3,5}&=\left\{ {u}{v}\in E({MIG}_{{\alpha },{\beta }}): {{d}}_{u}=3, {{d}}_{v}=5\right\} \\ {E}_{3,6}&=\left\{ {u}{v}\in E({MIG}_{{\alpha },{\beta }}): {{d}}_{u}=3, {{d}}_{v}=6\right\} . \end{aligned}$$

These edge partitions have a cardinality of $\left| {E}_{1,3}\right| =\left| {E}_{1,5}\right| =1,\left| {E}_{2,5}\right| =\left| {E}_{3,5}\right| =2, \left| {E}_{1,6}\right| ={\beta }+5, \left| {E}_{2,2}\right| =5, \left| {E}_{2,3}\right| =6, \left| {E}_{2,6}\right| =2\left( {\beta }+4\right) , \left| {E}_{3,3}\right| =3{\beta }+1,$ and $\left| {E}_{3,6}\right| =27{\beta }+7.$ Then from the definition 1.1, the modified polynomial of ${MIG}_{{\alpha },{\beta }}$ is:

30 $$\begin{aligned} M\left( {MIG}_{{\alpha },{\beta }};{x},{y}\right)&=\sum _{{i}\le {j}}{\alpha }_{{i},{j}}\left( {{MIG}_{{\alpha },{\beta }}}\right) {x}^{i}{y}^{j}\nonumber \\&=\left| {E}_{1,3}\right| \left( {{MIG}_{{\alpha },{\beta }}}\right) x^1y^3 + \left| {E}_{1,5}\right| \left( {{MIG}_{{\alpha },{\beta }}}\right) x^1y^5 + \left| {E}_{1,6}\right| \left( {{MIG}_{{\alpha },{\beta }}}\right) x^1y^6 \nonumber \\&\quad + \left| {E}_{2,2}\right| \left( {{MIG}_{{\alpha },{\beta }}}\right) x^2y^2 + \left| {E}_{2,3}\right| \left( {{MIG}_{{\alpha },{\beta }}}\right) x^2y^3 + \left| {E}_{2,5}\right| \left( {{MIG}_{{\alpha },{\beta }}}\right) x^2y^5 \nonumber \\ &\quad + \left| {E}_{2,6}\right| \left( {{MIG}_{{\alpha },{\beta }}}\right) x^2y^6 + \left| {E}_{3,3}\right| \left( {{MIG}_{{\alpha },{\beta }}}\right) x^3y^3 + \left| {E}_{3,5}\right| \left( {{MIG}_{{\alpha },{\beta }}}\right) x^3y^5 \nonumber \\ &\quad + \left| {E}_{3,6}\right| \left( {{MIG}_{{\alpha },{\beta }}}\right) x^3y^6,\nonumber \\&={x}\left[ {y}^3+{y}^5+\left( {\beta }+5\right) {y}^6\right] + {x}^2\left[ 5{y}^2+6{y}^3+2{y}^5+2\left( {\beta }+4\right) {y}^6\right] \nonumber \\ &\quad + {x}^3\left[ \left( 3{\beta }+1\right) {y}^3 + 2{y}^5 + \left( 27{\beta }+7\right) {y}^6\right] . \end{aligned}$$

$$\square$$

### Lemma 2.4

Let ${MIG}_{{\alpha },{\beta }}$ be the configuration of magnesium iodide, having ${\alpha }=2\left( {\beta }+1\right) ,$ ${\beta }\ge 1,$ Then the differential operators are

31 $$\begin{aligned} \Delta {x}\left[ M\left( {MIG}_{{\alpha },{\beta }};{x},{y}\right) \right]&={x}\left[ {y}^3+{y}^5+\left( {\beta }+5\right) {y}^6\right] + 2{x}^2\left[ 5{y}^2+6{y}^3+2{y}^5+2\left( {\beta }+4\right) {y}^6\right] \nonumber \\&\quad + 3{x}^3\left[ \left( 3{\beta }+1\right) {y}^3+2{y}^5+\left( 27{\beta }+7\right) {y}^6\right] . \end{aligned}$$

32 $$\begin{aligned} \Delta {y}\left[ M\left( {MIG}_{{\alpha },{\beta }};{x},{y}\right) \right]&={x}\left[ 3{y}^3+5{y}^4+6\left( {\beta }+5\right) {y}^6\right] + 2{x}^2\left[ 5{y}^2+9{y}^3+5{y}^5+6\left( {\beta }+4\right) {y}^6\right] \nonumber \\&\quad + {x}^3\left[ 3\left( 3{\beta }+1\right) {y}^3+10{y}^5 + 6\left( 27{\beta }+7\right) {y}^6\right] . \end{aligned}$$

### Proof

Differentiate equation (18) in terms of *x* and then product the attained value with *x*,  nw we have the differential operator $\Delta {x}\left[ M\left( {MIG}_{{\alpha },{\beta }};{x},{y}\right) \right] .$ Similarly, differentiate equation (18) in terms of *y* and now this time product the answer with *y*,  after simplification we will have the required operator value $\Delta {y}\left[ M\left( {MIG}_{{\alpha },{\beta }};{x},{y}\right) \right] .$ $\square$

### Lemma 2.5

Let ${MIG}_{{\alpha },{\beta }}$ be the configuration of magnesium iodide, having ${\alpha }=2{\beta }+1,$ ${\beta }\ge 1.$ Then the integral operators are

33 $$\begin{aligned} S_{x}\left[ M\left( {MIG}_{{\alpha },{\beta }};{x},{y}\right) \right]&={x}\left[ {y}^3+{y}^5+\left( {\beta }+5\right) {y}^6\right] + \frac{1}{2}{x}^2\left[ 5{y}^2+6{y}^3+2{y}^5+2\left( {\beta }+4\right) {y}^6\right] \nonumber \\&\quad + \frac{1}{3}{x}^3\left[ \left( 3{\beta }+1\right) {y}^3 + 2{y}^5 + \left( 27{\beta }+7\right) {y}^6\right] . \end{aligned}$$

34 $$\begin{aligned} S_{y}\left[ M\left( {MIG}_{{\alpha },{\beta }};{x},{y}\right) \right]&={x}\left[ \frac{1}{3}{y}^3+\frac{1}{5}{y}^5+\frac{\left( {\beta }+5\right) }{6}{y}^6\right] + {x}^2\left[ \frac{5}{2}{y}^2+2{y}^3+\frac{2}{5}{y}^5 + \frac{\left( {\beta }+4\right) }{3}{y}^6\right] \nonumber \\&\quad + {x}^3\left[ \frac{\left( 3{\beta }+1\right) }{3}{y}^3+\frac{2}{5}{y}^5 + \frac{\left( 27{\beta }+7\right) }{6}{y}^6\right] . \end{aligned}$$

### Proof

It is given in the Eq. (14), $S_{x}\left[ M\left( {MIG}_{{\alpha },{\beta }};{x},{y}\right) \right] =\int _{0}^{x}\frac{M\left( {MIG}_{{\alpha },{\beta }};{t},{y}\right) }{t}dt.$ Now evaluate the equation (18) which is the methodology of the general modified polynomial for the structure of ${MIG}_{{\alpha },{\beta }}.$ Following a few mathematical calculations, we will have $S_{x}\left[ M\left( {MIG}_{{\alpha },{\beta }};{x},{y}\right) \right] .$ Similarly, from equation (15), $S_{y}\left[ M\left( {MIG}_{{\alpha },{\beta }};{x},{y}\right) \right] =\int _{0}^{y}\frac{M\left( {MIG}_{{\alpha },{\beta }};{x},{t}\right) }{t}dt.$ Now evaluate the equation (18) which is the methodology of the general modified polynomial for the structure of ${MIG}_{{\alpha },{\beta }}.$ Following a few mathematical calculations, we will have $S_{y}\left[ M\left( {MIG}_{{\alpha },{\beta }};{x},{y}\right) \right] .$ $\square$

### Lemma 2.6

Let ${MIG}_{{\alpha },{\beta }}$ be the configuration of magnesium iodide, having ${\alpha }=2\left( {\beta }+1\right) ,$ ${\beta }\ge 1.$ Then the *J* operator for the equation 29 is

$$\begin{aligned} {J}\left[ M\left( {MIG}_{{\alpha },{\beta }};{x},{y}\right) \right]&=6{x}^4+6{x}^5+\left( 3{\beta }+2\right) {x}^6+\left( {\beta }+7\right) {x}^7+2\left( {\beta }+5\right) {x}^8+\left( 27{\beta }+7\right) {x}^9. \end{aligned}$$

### Proof

We obtain the by applying the operator mentioned in the equation 16 on the fundamental equation described in 29. ${J}\left[ M\left( {MIG}_{{\alpha },{\beta }};{x},{y}\right) \right] .$ $\square$

### Theorem 2.7

Let ${MIG}_{{\alpha },{\beta }}$ be the configuration of magnesium iodide, having ${\alpha }=2{\beta }+1,$ ${\beta }\ge 1,$ and ${P}_{M_1}$ and ${P}_{M_2}$ are the “modified polynomial of first and second Zagreb indices”. Then ${P}_{M_1}\left( {{MIG}_{{\alpha },{\beta }}}\right)$ and ${P}_{M_2}\left( {{MIG}_{{\alpha },{\beta }}}\right)$ is

35 $$\begin{aligned} {P}_{M_1}\left( {{MIG}_{{\alpha },{\beta }}}\right)&={x}\left[ 4{y}^3+6{y}^5+7\left( {\beta }+5\right) {y}^6\right] + 2{x}^2\left[ 10{y}^2+15{y}^3+7{y}^5+8\left( {\beta }+4\right) {y}^6\right] \nonumber \\&\quad + {x}^3\left[ 9\left( 3{\beta }+1\right) {y}^3+12{y}^5+7\left( 27{\beta }+7\right) {y}^6\right] . \end{aligned}$$

36 $$\begin{aligned} {P}_{M_2}\left( {{MIG}_{{\alpha },{\beta }}}\right)&={x}\left[ 3{y}^3+5{y}^4+6\left( {\beta }+5\right) {y}^6\right] + 2{x}^2\left[ 10{y}^2+18{y}^3+10{y}^5+12\left( {\beta }+4\right) {y}^6\right] \nonumber \\ &\quad + 3{x}^3\left[ 3\left( 3{\beta }+1\right) {y}^3+10{y}^5+6\left( 27{\beta }+7\right) {y}^6\right] . \end{aligned}$$

### Proof

The technique of the modified polynomial of first Zagreb index given in the Definition 1.2, this methodology can be written as ${P}_{M_1}({{MIG}_{{\alpha },{\beta }}})=\left( \Delta {x}+\Delta {y}\right) \left( M\left( {{MIG}_{{\alpha },{\beta }}};{x},{y}\right) \right) ,$ for ${MIG}_{{\alpha },{\beta }}$-structure. Now evaluating the differential operators described in Lemma 2.1, for the structure of ${MIG}_{{\alpha },{\beta }}.$ After some algebraic simplifications we will get the modified polynomial of first Zagreb index for ${MIG}_{{\alpha },{\beta }}$ as:

$${P}_{M_1}\left( {{MIG}_{{\alpha },{\beta }}}\right) ={x}\left[ 4{y}^3+6{y}^5+7\left( {\beta }+5\right) {y}^6\right] + 2{x}^2\left[ 10{y}^2+15{y}^3+7{y}^5+8\left( {\beta }+4\right) {y}^6\right] + {x}^3\left[ 9\left( 3{\beta }+1\right) {y}^3+12{y}^5+7\left( 27{\beta }+7\right) {y}^6\right] .$$

Similarly evaluating the differential operators, given in the Lemma 2.1 for the structure of ${MIG}_{{\alpha },{\beta }}$ in ${P}_{M_2}({{MIG}_{{\alpha },{\beta }}})=\left( \Delta {x}\Delta {y}\right) \left( M\left( {{MIG}_{{\alpha },{\beta }}};{x},{y}\right) \right) ,$ and Following a few mathematical calculations, we are going to accomplish the desired outcomes of modified polynomial of second Zagreb index for ${MIG}_{{\alpha },{\beta }}$:

$${P}_{M_2}\left( {{MIG}_{{\alpha },{\beta }}}\right) ={x}\left[ 3{y}^3+5{y}^4+6\left( {\beta }+5\right) {y}^6\right] + 2{x}^2\left[ 10{y}^2+18{y}^3+10{y}^5+12\left( {\beta }+4\right) {y}^6\right] + 3{x}^3\left[ 3\left( 3{\beta }+1\right) {y}^3+10{y}^5+6\left( 27{\beta }+7\right) {y}^6\right] .$$ $$\square$$

### Theorem 2.8

Let ${MIG}_{{\alpha },{\beta }}$ be the configuration of magnesium iodide, having ${\alpha }=2{\beta }+1,$ ${\beta }\ge 1,$ and ${P}_{^{m}M_2}$ is the “modified polynomial of second modified Zagreb index”. Then ${P}_{^{m}M_2}\left( {{MIG}_{{\alpha },{\beta }}}\right)$ is

37 $$\begin{aligned} {P}_{^{m}M_2}({{MIG}_{{\alpha },{\beta }}})&={x}\left[ \frac{1}{3}{y}^3+\frac{1}{5}{y}^5+\frac{\left( {\beta }+5\right) }{6}{y}^6\right] + 2{x}^2\left[ \frac{5}{2}{y}^2+2{y}^3+\frac{2}{5}{y}^5+\frac{\left( {\beta }+4\right) }{3}{y}^6\right] \nonumber \\&\quad + 3{x}^3\left[ \frac{3{\beta }+1}{3}{y}^3+\frac{2}{5}{y}^5+\frac{\left( 27{\beta }+7\right) }{6}{y}^6\right] . \end{aligned}$$

### Proof

The technique of the modified polynomial of second modified Zagreb given in the Definition 1.3, this methodology can be written as ${P}_{^{m}M_2}({{MIG}_{{\alpha },{\beta }}})=\left( S_{x}S_{y}\right) \left( M\left( {{MIG}_{{\alpha },{\beta }}};{x},{y}\right) \right) ,$ for ${MIG}_{{\alpha },{\beta }}$-structure. Now evaluating the integral operators described in Lemma 2.2, for the structure of ${MIG}_{{\alpha },{\beta }}.$ Following a few mathematical calculations, we are going to accomplish the desired outcome of modified polynomial of second modified Zagreb index for ${MIG}_{{\alpha },{\beta }}$ as:

$${P}_{^{m}M_2}\left( {{MIG}_{{\alpha },{\beta }}}\right) ={x}\left[ \frac{1}{3}{y}^3+\frac{1}{5}{y}^5+\frac{\left( {\beta }+5\right) }{6}{y}^6\right] + 2{x}^2\left[ \frac{5}{2}{y}^2+2{y}^3+\frac{2}{5}{y}^5+\frac{\left( {\beta }+4\right) }{3}{y}^6\right] + 3{x}^3\left[ \frac{3{\beta }+1}{3}{y}^3+\frac{2}{5}{y}^5+\frac{\left( 27{\beta }+7\right) }{6}{y}^6\right] .$$ $$\square$$

### Theorem 2.9

Let ${MIG}_{{\alpha },{\beta }}$ be the configuration of magnesium iodide, having ${\alpha }=2{\beta }+1,$ ${\beta }\ge 1,$ and ${P}_{R_{\alpha }}$ is the “modified polynomial of general Randić index”. Then ${P}_{R_{\alpha }}\left( {{MIG}_{{\alpha },{\beta }}}\right)$ is

38 $$\begin{aligned} {P}_{R_{\alpha }}({{MIG}_{{\alpha },{\beta }}})&={x}\left[ 3^{\alpha }{y}^3+5^{\alpha }{y}^5+6^{\alpha }\left( {\beta }+5\right) {y}^6\right] + 2^{{\alpha }}{x}^2\left[ 5\times 2^{\alpha }{y}^2+6\times 3^{\alpha }{y}^3+2\times 5^{\alpha }{y}^5\right. \nonumber \\&\quad \left. +2\times 6^{\alpha }\left( {\beta }+4\right) {y}^6\right] + 3^{\alpha }{x}^3\left[ 3^{{\alpha }}\left( 3{\beta }+1\right) {y}^3+2\times 5^{\alpha }{y}^5+6^{\alpha }\left( 27{\beta }+7\right) {y}^6\right] . \end{aligned}$$

### Proof

The technique defined in the Definition 1.4 is known as the formula for the modified polynomial of general Randić index for ${MIG}_{{\alpha },{\beta }}$ defined as: ${P}_{R_{\alpha }}({{MIG}_{{\alpha },{\beta }}})=\left( D^{\alpha }_{x}D^{\alpha }_{y}\right) \left( M\left( {{MIG}_{{\alpha },{\beta }}};{x},{y}\right) \right) ,$ by evaluating the formula of generalize version of differential operators given by Lemma 2.1 for ${MIG}_{{\alpha },{\beta }}.$ Following a few mathematical calculations, we are going to accomplish the desired outcomes of modified polynomial of general Randić index for ${MIG}_{{\alpha },{\beta }}$ as:

$${P}_{R_{\alpha }}\left( {{MIG}_{{\alpha },{\beta }}}\right) = {x}\left[ 3^{\alpha }{y}^3+5^{\alpha }{y}^5+6^{\alpha }\left( {\beta }+5\right) {y}^6\right] + 2^{{\alpha }}{x}^2\left[ 5\times 2^{\alpha }{y}^2+6\times 3^{\alpha }{y}^3+2\times 5^{\alpha }{y}^5+2\times 6^{\alpha }\left( {\beta }+4\right) {y}^6\right]$$

$$+ 3^{\alpha }{x}^3\left[ 3^{{\alpha }}\left( 3{\beta }+1\right) {y}^3+2\times 5^{\alpha }{y}^5+6^{\alpha }\left( 27{\beta }+7\right) {y}^6\right] .$$ $$\square$$

### Theorem 2.10

Let ${MIG}_{{\alpha },{\beta }}$ be the configuration of magnesium iodide, having ${\alpha }=2{\beta }+1,$ ${\beta }\ge 1,$ and ${P}_{R_{\alpha }}$ is the “modified polynomial of general inverse Randić index”. Then ${P}_{IR_{\alpha }}\left( {{MIG}_{{\alpha },{\beta }}}\right)$ is

39 $$\begin{aligned} {P}_{IR_{\alpha }}({{MIG}_{{\alpha },{\beta }}})&={x}\left[ \frac{1}{3^{\alpha }}{y}^3+\frac{1}{5^{\alpha }}{y}^5+\frac{\left( {\beta }+5\right) }{6^{\alpha }}{y}^6\right] + \frac{1}{2^{{\alpha }}}{x}^2\left[ \frac{5}{2^{\alpha }}{y}^2+\frac{6}{3^{\alpha }}{y}^3+\frac{2}{5^{\alpha }}{y}^5+\frac{2\left( {\beta }+4\right) }{6^{\alpha }}{y}^6\right] \nonumber \\ &\quad + \frac{1}{3^{\alpha }}{x}^3\left[ \frac{3{\beta }+1}{3^{{\alpha }}}{y}^3+\frac{2}{5^{\alpha }}{y}^5+\frac{\left( 27{\beta }+7\right) }{6^{\alpha }}{y}^6\right] . \end{aligned}$$

### Proof

The technique defined in the Definition 1.4 is known as the formula for the modified polynomial of general inverse version of Randić index for ${MIG}_{{\alpha },{\beta }}$ defined as: ${P}_{IR_{\alpha }}({{MIG}_{{\alpha },{\beta }}})=\left( S^{\alpha }_{x}S^{\alpha }_{y}\right) \left( M\left( {{MIG}_{{\alpha },{\beta }}};{x},{y}\right) \right) ,$ by evaluating the formula of generalize version of integral operators given by Lemma 2.2 for ${MIG}_{{\alpha },{\beta }}.$ Following a few mathematical calculations, we are going to accomplish the desired outcomes of modified polynomial of general inverse version of Randić index for ${MIG}_{{\alpha },{\beta }}$ as:

$${P}_{IR_{\alpha }}\left( {{MIG}_{{\alpha },{\beta }}}\right) = {x}\left[ \frac{1}{3^{\alpha }}{y}^3+\frac{1}{5^{\alpha }}{y}^5+\frac{\left( {\beta }+5\right) }{6^{\alpha }}{y}^6\right] + \frac{1}{2^{{\alpha }}}{x}^2\left[ \frac{5}{2^{\alpha }}{y}^2+\frac{6}{3^{\alpha }}{y}^3+\frac{2}{5^{\alpha }}{y}^5+\frac{2\left( {\beta }+4\right) }{6^{\alpha }}{y}^6\right] + \frac{1}{3^{\alpha }}{x}^3\left[ \frac{3{\beta }+1}{3^{{\alpha }}}{y}^3+\frac{2}{5^{\alpha }}{y}^5+\frac{\left( 27{\beta }+7\right) }{6^{\alpha }}{y}^6\right] .$$ $$\square$$

## Importance of computing modified polynomials of Magnesium Iodide Structure

Calculating modified polynomials for the crystal structure of magnesium iodide (MgI2) can provide useful information on the material’s characteristics, behavior, and prospective uses. In quantitative structure-property relationship (QSPR) investigations, modified polynomials are utilized to establish correlations between structural traits and other attributes^46–49^. Here are some reasons why finding modified polynomials for the magnesium iodide structure is important:

*Property Prediction:* Based on the crystal structure, modified polynomials may be used to forecast a wide variety of material attributes. Electronic, optical, thermal, and mechanical qualities are all included. Researchers can create prediction models that help in property estimate by comparing the structural traits recorded by the modified polynomials with experimental or theoretical property data. *Structure-Property Relationships:* Researchers can discover key structure-property correlations by computing modified polynomials and studying their interactions with various properties. This understanding can help guide the design and development of magnesium iodide-based materials for specific applications such semiconductors, optoelectronic devices, and solid-state electrolytes.

*Material Design and Optimization:* modified polynomials give a systematic method for investigating the effect of structural modifications on material characteristics. These polynomials can help researchers develop novel materials with specific characteristics by altering the crystal structure or introducing flaws or replacements. *Understanding Phase Transitions:* Modified polynomials can help identify structural changes that accompany these transitions, shed light on the underlying mechanisms, and offer understandings into the stability and behavior of various phases. *Characterization of Defects:* Point flaws like voids or interstitials can significantly alter the properties of a material. To measure the impact of faults on crystal structure and properties, which is essential for comprehending material performance and dependability, modified polynomials can be used.

*High-Throughput Screening:* Modified polynomials can be used to quickly assess materials’ potential properties during high-throughput screening. This is particularly helpful in the field of materials research, as experts look for promising candidates among a vast array of potential uses. *Accelerating Research and Development:* Modified polynomials provide a systematic and quantitative technique to relate structure and properties, which speeds up the research and development of magnesium iodide-based materials. In the domains of electronics, energy storage, and materials engineering in particular, this is advantageous. *Comparative Analysis:* Magnesium iodide can be compared to other materials using modified polynomials. This can help researchers find similarities and differences in structural motifs and property trends, which will help them better grasp the principles of material design.

To summarize, computing modified polynomials for the crystal structure of magnesium iodide provides a methodical and data-driven way to understanding its features and prospective uses. modified polynomials contribute to the efficient design, development, and assessment of magnesium iodide-based materials for diverse technical and scientific uses by creating connections between structure and characteristics. For further detail on the topic, we refer to see^50–52^.

## Applications of computing modified polynomials of Magnesium Iodide Structure

*Material Science and Chemistry:* Understanding the modified polynomials of the magnesium iodide structure can provide insights into its electrical structure, bonding, and stability. This knowledge can be used in material design and synthesis. *Electronic Structure Analysis:* This research could be utilized to investigate the electronic characteristics of magnesium iodide, which could lead to the creation of improved electronic materials or semiconductors. *Energy Storage and Conversion:* Knowledge of the modified polynomials could be used to build materials for energy storage systems (batteries and capacitors) or energy conversion devices (solar cells).

*Catalysis:* Understanding the structural changes in magnesium iodide could have significance in catalysis, particularly in heterogeneous catalysis, where surface features are important. *Crystallography and X-ray Diffraction:* Crystallography and X-ray diffraction techniques, which are used to determine the atomic structure of materials, could benefit from the updated polynomials. *Quantum Chemistry and Computational Modeling:* Researchers in quantum chemistry and computer modeling can utilize modified polynomials to refine theoretical models and improve simulation accuracy.

*Chemical Sensors:* Understanding the structure of magnesium iodide may lead to the development of chemical sensors capable of detecting certain chemicals or ions, which might be beneficial in environmental monitoring or medical diagnostics. *Pharmaceuticals:* Because crystallography is used to determine the structure of medicinal molecules, understanding the structural alterations in magnesium iodide can have significance for drug development.

*Educational and Research Purposes:* Modified polynomials in magnesium iodide can be used as educational material in chemistry and materials science courses, as well as give research opportunities for students and scientists. *Interdisciplinary Research:* The discoveries have the potential to ignite interdisciplinary cooperation among chemists, physicists, materials scientists, and engineers, leading to novel applications and technologies.

Finally, study on the modified polynomials of the magnesium iodide structure has the potential to contribute to a wide range of scientific and practical domains, including material science, electronic devices, and beyond.

## Conclusion

A structure’s modified polynomial provides the polynomial or abstracted function that describes a chemical structure or network. For the magnesium iodide or $Mgl_2$ structure, we computed the modified polynomials of the first, second, and modified Zagreb indexes, as well as the general and inverse Randić indexes, for both even and odd occurrences of parameter $\alpha .$ Certain algebraic properties of the magnesium iodide structure were added up.

## Data Availability

The datasets used and/or analysed during the current study available from the corresponding author on reasonable request.
